# Solvent-Free Catalytic Oxidation of Benzyl Alcohol over Au-Pd Bimetal Deposited on TiO_2_: Comparison of Rutile, Brookite, and Anatase

**DOI:** 10.1186/s11671-019-3211-8

**Published:** 2019-12-27

**Authors:** Xiaoliang Li, Jiangjiang Feng, Jia Sun, Zhe Wang, Wei Zhao

**Affiliations:** 1 State Key Laboratory Breeding Base of Coal Science and Technology Co-founded by Shanxi Province and the Ministry of Science and Technology, Taiyuan University of Technology, Taiyuan, 030024 Shanxi People’s Republic of China; 2 Cardiff Catalysis Institute, School of Chemistry, Cardiff University, Cardiff, CF10 3AT UK

**Keywords:** Au-Pd nanoparticles, TiO_2_ crystal form, Brookite, Rutile, Anatase, Benzyl alcohol oxidation, Solvent-free

## Abstract

TiO_2_ (P25)-supported Au-Pd bimetal nanoparticles displayed excellent performance in the solvent-free benzyl alcohol catalytic oxidation. However, little research attention has been paid to investigate the effects of TiO_2_ form on the catalytic activity of Au-Pd/TiO_2_. In the present research, rutile, brookite, and anatase TiO_2_ were successfully synthesized and subsequently applied as the carrier to load Au-Pd nanoparticles by the deposition-precipitation method. The experimental results indicated that the benzyl alcohol conversion employing the rutile TiO_2_-supported Au-Pd catalyst is higher than the conversion of anatase and brookite TiO_2_-loaded Au-Pd catalysts. However, the Au-Pd/TiO_2_-rutile displayed the lowest and highest selectivity toward benzaldehyde and toluene, respectively. ICP-AES, XRD, XPS, and TEM were conducted to characterize these catalysts. The corresponding experimental results revealed that the excellent performance of Au-Pd/TiO_2_-rutile catalyst was attributed to both the smaller Au-Pd nanoparticle size distribution and the higher concentrations of O_α_ and Pd^2+^ species on the catalyst surface. In the recycle experiments, the Au-Pd/TiO_2_-rutile catalyst displayed lower reaction stability compared with the Au-Pd/TiO_2_-anatase and Au-Pd/TiO_2_-brookite, which might be due to the coverage of larger amount of aldehyde products on the surface.

## Introduction

Gold has long been considered to be chemically inert until Hutchings and Haruta independently discovered their excellent catalytic activities in acetylene hydrochlorination and low-temperature CO catalytic oxidation, respectively [1, 2]. Gold, as the active component, has been widely studied in many reactions, including water-gas shift reaction, the direct synthesis of H_2_O_2_ from O_2_ and H_2_, and the selective hydrogenation of cinnamaldehyde [3–5]. Besides, the reactivity and stability of Au-based catalysts could be significantly enhanced by combining them with Pd. For example, Hutchings et al. found that the catalytic activity of Au-Pd bimetal immobilized on TiO_2_ (P25) is much higher than the supported Au or Pd catalysts for the benzyl alcohol oxidation in the absence of solvent [6].

It is generally accepted that the catalytic activity of the supported Au-Pd bimetal catalyst is closely related to the property of support, the nanoparticle size, and the preparation method. As the catalyst supports, reducible metal oxides such as TiO_2_, CeO_2_, and Fe_2_O_3_ have been widely adopted due to the strong interaction between the metals and carrier, together with the facile activation of molecular oxygen. TiO_2_, as the typical carrier candidate, has been extensively studied for supporting Au-Pd, which also showed the outstanding catalytic activity for the benzyl alcohol oxidation. For example, Hutchings et al. firstly prepared the Au-Pd/TiO_2_ by the sol-immobilization method and applied for the selective catalytic oxidation of alcohols into aldehydes. The corresponding results indicated that alloying Au with Pd can result in up to a twenty-five-fold enhancement in the activity compared with the Au catalyst, while retaining the selectivity [6]. Chadwick et al. recently prepared the bimetallic Au-Pd/TiO_2_ nanotubes by using colloidal synthesis and immobilization on sodium-free Ti-nanotubes, which displayed superior catalytic performance for the oxidation of benzyl alcohol to benzaldehyde [7]. Zheng et al. developed a series of Au@Pd/TiO_2_ catalysts with highly dispersed Pd by a two-step photo-deposition method, which also acted as a high active catalyst for benzyl alcohol aerobic oxidation under solvent-free conditions [8]. Li et al. adopted bio-reductive approach with cacumen platycladi extract to fabricate Au-Pd/TiO_2_ and then applied it into solvent-free oxidation of benzyl alcohol. The catalyst displayed excellent catalytic performance, durability, and reusability [9].

The most widely used type of TiO_2_ is P25. However, it is recognized that titania contains three different forms: rutile, brookite, and anatase. Up to now, it could be seen that the influence of TiO_2_ form on the catalytic activity has been reported only in photocatalytic reactions and gas-solid catalytic oxidation reactions, such as CO_2_ photocatalytic reduction, CO catalytic oxidation, and NH_3_ selective catalytic reduction NO_*x*_ [10–12]. It is universally acknowledged that anatase TiO_2_, as a carrier, usually exhibits better catalytic activity than that of rutile and brookite TiO_2_. Nevertheless, this viewpoint is contrary to some experimental results. Dai et al. used deposition-precipitation method to load Au onto the surface of anatase, rutile, brookite TiO_2_, and P25, respectively and investigated their catalytic activity for CO oxidation. The corresponding results suggested that the brookite TiO_2_-supported gold catalyst sustains the highest catalytic activity due to the smaller Au nanoparticles on the surface [11]. Yao et al. prepared CeO_2_/anatase, CeO_2_/brookite, and CeO_2_/rutile with the traditional incipient-wetness impregnating method and compared their NH_3_-SCR catalytic performance. The experimental results indicated that the optimal catalytic performance could be obtained from the CeO_2_/rutile, which could be due to its excellent redox performance, higher concentration of acidic sites, Ce^3+^ species, and adsorbed oxygen species on the catalyst surface [12]. Li et al. studied the CO_2_ photoreduction with water vapor over rutile, anatase, and brookite TiO_2_; the experimental results demonstrated that brookite TiO_2_ showed the better photocatalytic performance compare with the anatase and rutile, which is related to the lowest formation energy barrier of oxygen vacancy on the brookite surface [10].

Although Au-Pd/TiO_2_ catalyst has shown the outstanding catalytic activity in benzyl alcohol oxidation, the influence of the TiO_2_-supported Au-Pd form on the catalytic performance has not been examined up to the present. Hence, it is essential to perform comparative experiments to investigate the differences in catalytic activity and reveal the reasons for the different catalytic performance of TiO_2_-supported Au-Pd on rutile, brookite, and anatase TiO_2_. The present work studied the catalytic activity of benzyl alcohol oxidation over bimetal Au-Pd supported on anatase, rutile, and brookite, respectively. Meanwhile, XRD, ICP-AES, XPS, and TEM were applied to reveal the effect of TiO_2_ form on the physicochemical properties of Au-Pd/TiO_2_.

## Methods

All chemical reagents were purchased from Aladdin Company (Shanghai China) and used as received: Urea (99.9% metal basis), titanium bis (ammonium lactate) dihydroxide aqueous solution (TBD, 50% in water ), TiCl_4_ (99.99% metals basis), ethanol (≥ 99.5%, purity), H_2_SO_4_ (> 98%, purity), PdCl_2_ (99.99% metals basis), HAuCl_4_·3H_2_O (≥ 99.9% trace metals basis), benzyl alcohol (99.8%, purity). O_2_ (99.999%, purity) was supplied from Taiyuan Iron and Steel corporation.

### Synthesis of Brookite and Anatase TiO_2_ [13]

To prepare the brookite TiO_2_, 8 mL of TBD solution (50%) and 17 g urea were first mixed, then the mixture solution was adjusted to 80 mL by the addition of the extra deionized water. Subsequently, the resulting solution was transferred into a 200-mL Teflon-lined autoclave, which was encapsulated and kept at 160 °C for 24 h. When the autoclave was cooled to room temperature, the precipitates were filtered, washed, and dried. Finally, the obtained powder was calcined at 500 °C for 5 h.

For the preparation of anatase TiO_2_, we only adjusted the dosage of urea from 17 to 0.48 g and repeated the steps as indicated above.

### Synthesis of Rutile TiO_2_ [10]

For the rutile TiO_2_, the required amount of TiCl_4_ was dissolved into ethanol under stirring. After the yellowish sol formed, water was added into the above solution, drop by drop, while stirring. The molar ratio of TiCl_4_, ethanol, and water was controlled at 2:20:280. The resulting mixture was stirred for another 3 h and aged at 50 °C for 24 h in a well-closed autoclave. Subsequently, the white precipitate was centrifuged, washed, and dried. Finally, the obtained product was also calcined at 500 °C for 5 h.

### Preparation of Au-Pd Deposited on Brookite, Anatase, and Rutile

To keep the Au:Pd molar ratio of 1:1, the nominal loadings of Au and Pd on the Au-Pd/TiO_2_ catalysts were 1.00 wt% and 0.54 wt%, respectively. 1.00 wt% Au-0.54 wt% Pd/TiO_2_ (brookite, anatase, and rutile) were prepared by the deposition-precipitation method, with urea as a precipitant. Typically, for the preparation of Au-Pd/TiO_2_-brookite, 2 mL aqueous solution of HAuCl_4_ (5 mg Au/mL), 1.08 mL aqueous solution of PdCl_2_ (5 mg Pd/mL), 0.985 g brookite TiO_2_, and 3.48 g urea were added into 100 mL deionized water under stirring at room temperature. The mixture solution was stirred at 80 °C for 6 h. Then, the obtained solution was aged at room temperature for another 12 h. Subsequently, the precipitate was centrifuged, washed, and dried. Finally, the obtained product was calcined at 300 °C for 2 h with the heating rate of 2 °C/min.

For simplicity, the prepared TiO_2_-brookite, TiO_2_-rutile, TiO_2_-anatase catalysts Au-Pd/TiO_2_-brookite, Au-Pd/TiO_2_-rutile, and Au-Pd/TiO_2_-anatase samples were denoted as TiO_2_-B, TiO_2_-R, TiO_2_-A, ATB, ATR, and ATA, respectively.

### Benzyl Alcohol Oxidation

The benzyl alcohol catalytic oxidation was performed in a mechanically stirred reactor using 50-mL glass-lined min claves (Anhui Kemi machinery Technology Co., Ltd, China). Typically, 15 mL benzyl alcohol and 0.05 g catalyst were introduced into the reactor, and the reactor was sealed and purged for 5 times by O_2_. Subsequently, the reactor was pressurized to 0.3 MPa with O_2_ at room temperature. The reaction mixture was heated to the required temperature at 1000 rpm. The reactor was also connected with the oxygen reservoir for the purpose of replenishing the consumed oxygen during the reaction. The reaction products were analyzed by GC (FuLi GC9790, Zhejiang, China) equipped with a flame ionization detector (FID) and a DM-5 column (30 m × 0.25 mm × 0.25 μm). In order to ensure the reliability of the data, each group of experiments was repeated at least twice, and every data point was determined three times by GC.

To investigate the stability of the catalytic activity, the reused catalyst was performed within three catalytic circles. After each run, the catalyst was collected and washed with acetone and then heated at 80 °C for 16 h.

### Characterization

Powder XRD was performed on a Rigaku D/max-RC diffractometer with CuKα radiation at 40 kV and 25 mA (*λ* = 0.15418 nm). The intensities were recorded within the scanning range of 10–90° at the speed of 8 °/min. ICP-AES was conducted to quantitatively determine the chemical composition of the prepared catalysts on an Agilent 735-ES instrument. Before measurements, the catalyst was dissolved in aqua regia for about 24 h. X-ray photoelectron spectroscopy measurements were carried out on a PHI-1600ESCA System XPS spectrometer (Perkin-Elmer, USA) using non-monochromatic Mg-Kα radiation, operating at 15 kV and under 10^−7^ Pa pressure with photoelectron energy set at 1254 eV. The reported binding energies were referenced to the C1s binding energy of 284.6 eV. TEM was performed on a JEM-2100 electron microscope, operating at 200 kV. Before analysis, ethanol was used to disperse the sample powders with the assistance of the ultrasound and then the mixed solution was deposited onto a mesh grid with carbon film.

## Results and Discussion

**Fig. 1:**
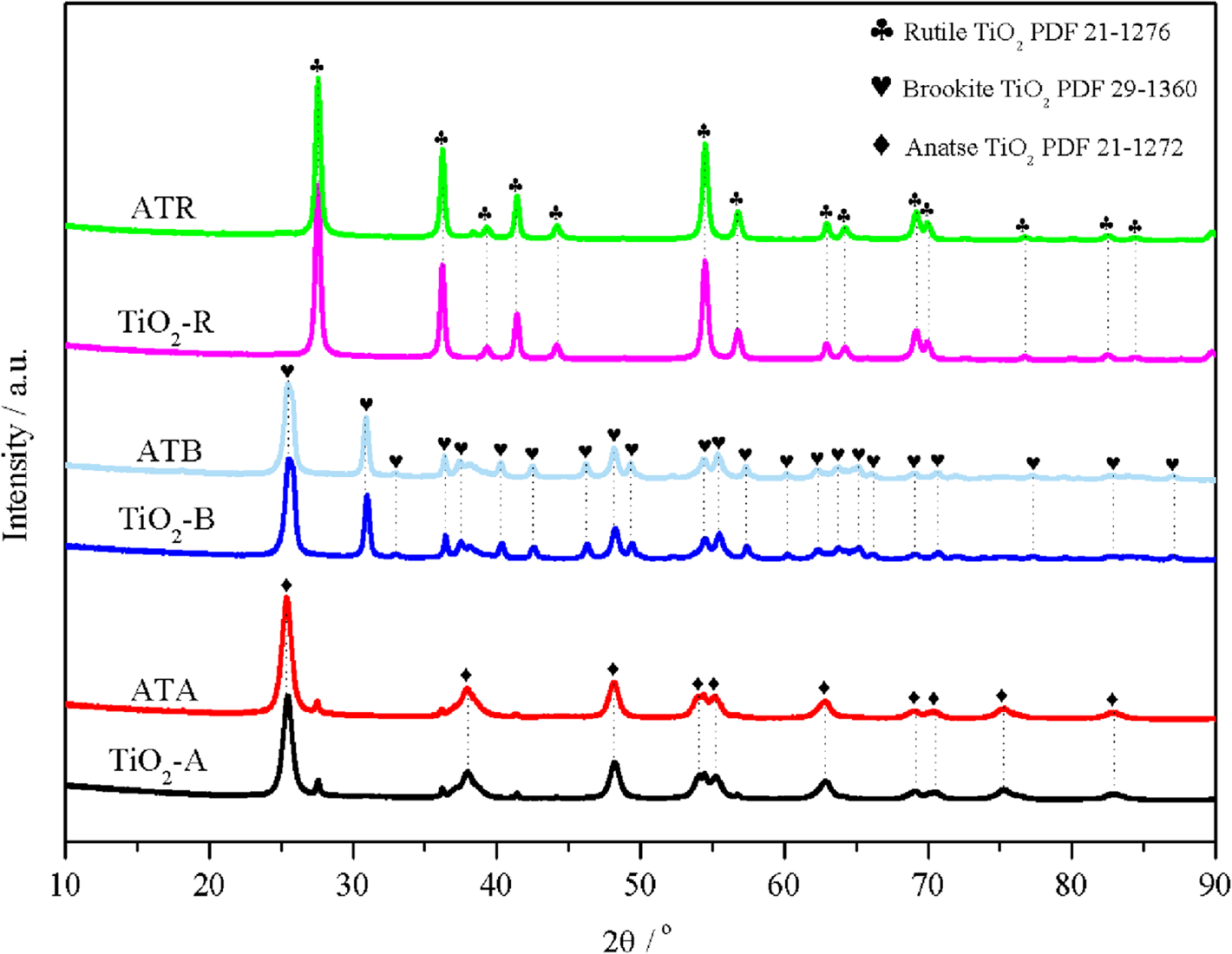
XRD patterns of the TiO_2_-A, ATA, TiO_2_-B, ATB, TiO_2_-R, and ATR samples

XRD was performed to investigate the crystal form of the TiO_2_ carriers and the Au-Pd dispersion states on the Au-Pd/TiO_2_ catalysts. As shown in Fig. 1, ten diffraction peaks at 25.4, 37.8, 48.1, 54.1, 55.2, 62.9, 68.8, 70.4, 75.1, and 82.7° were detected for the prepared anatase TiO_2_, which were consistent with the standard PDF card (No. 21-1272). It could also be found that a series of peaks at 25.4, 30.9, 32.8, 36.3, 37.4, 40.2, 42.4, 46.2, 48.2, 49.3, 54.4, 55.3, 57.4, 60.2, 62.2, 63.8, 65.1, 66.1, 69.1, 70.7, 77.2, 82.7, and 87.0° were detected on the prepared brookite TiO_2_ carrier, which fit well with the standard brookite PDF card (brookite TiO_2_ PDF 29-1360). The prepared rutile TiO_2_ exhibited the corresponding peaks at 27.5, 36.1, 39.2, 41.3, 44.2, 54.3, 56.6, 62.7, 64.1, 69.1, 69.9, 76.6, 82.4, and 84.3°, which was also consistent with the rutile TiO_2_ PDF card (rutile TiO_2_ PDF 21-1276). The above XRD results confirmed that TiO_2_ with rutile, brookite, and anatase forms were successfully prepared. Meanwhile, the average TiO_2_ crystallite sizes were estimated by using the Scherrer equation based on the information of diffraction peaks at different locations (*2θ* = 25.4° for TiO_2_-A, 30.9° for TiO_2_-B, and 27.6° for TiO_2_-R); the calculated results showed that the nanoparticle sizes of TiO_2_ were arranged in the following sequence: TiO_2_-R (27.6 nm) > TiO_2_-B (18.9 nm) > TiO_2_-A(11.2 nm). After loading of Au-Pd bimetal nanoparticles on the surfaces of TiO_2_ carriers, no diffraction peaks assigned to Au or Pd were detected on the prepared Au-Pd/TiO_2_ patterns. This phenomenon not only indicated that Au and Pd were highly dispersed into smaller particle sizes (e.g. 3~5 nm), which cannot be observed by the XRD, but also suggested that the crystalline structure of the TiO_2_ carriers was unaffected by the loading of Au and Pd.

To determine the actual contents of Au and Pd on the prepared Au-Pd/TiO_2_ catalysts, the ICP-AES was conducted. The corresponding results are listed in Table 1. It was found that the actual bulk metal concentrations were lower than the nominal values, which might have been caused by the leaching of weakly adsorbed Au-Pd nanoparticles during the filtration or washing process.

**Table 1:** Au and Pd bulk composition on the Au-Pd/TiO_2_ catalysts

Catalysts	Au (wt%)		Pd (wt%)		Au/Pd molar ratio
	Nominal	Actual	Nominal	Actual	
ATB	1.00	0.94	0.54	0.45	1.13
ATR	1.00	0.84	0.54	0.45	1.01
ATA	1.00	0.94	0.54	0.49	1.04

**Fig. 2:**
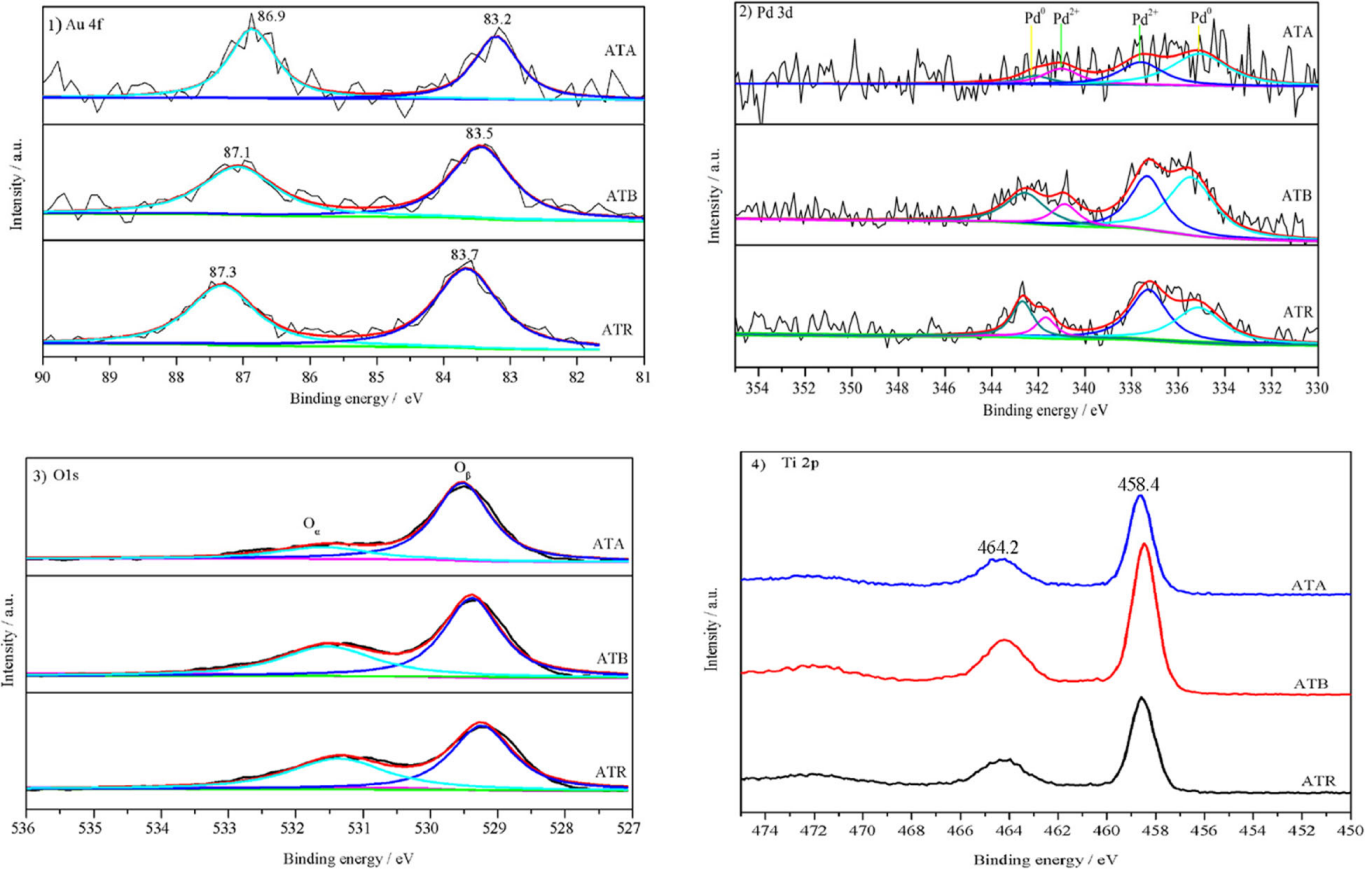
XPS spectra of (**1**) Au 4f , (**2**)Pd 3d, (**3**) O1s, and (**4**) Ti 2p) for the ATA, ATB, and ATR samples

XPS, as a surface-sensitive probe technique, was carried out to detect the surface element composition and the chemical states. Figure 2 displayed the Au (4f), Pd (3d), O (1 s), and Ti (2p) spectra for the Au-Pd nanoparticles supported on TiO_2_ catalysts. As shown in Fig. 2.1, Au 4f spectra were detected at two locations on each catalyst, but the specific positions of the two peaks were slightly different. It is generally acknowledged that the metallic state Au 4f spectrum usually displays two contributions (4f_7/2_ and Au 4f_5/2_), which were located at 84.0 and 87.7 eV, respectively [14]. The observed Au 4f spectra negative shift for these three Au-Pd/TiO_2_ catalysts could be explained by the electronic modification of Au species by Pd species, which also suggests the strong interaction between Au and Pd species. Furthermore, no ionic Au species were detected on the prepared Au-Pd/TiO_2_ catalysts.

Figure 2.2 showed the XPS spectra of the Pd 3d core level regions of the ATA, ATB, and ATR samples. According to the previous reports, the bands of Pd 3d could be deconvoluted into four sub-peaks; the Pd 3d_3/2_ and 3d_5/2_ peaks at around 335 and 341 eV are attributed to the metallic Pd^0^ [15], the Pd 3d_5/2_ and 3d_7/2_ centered at about 337 and 342 eV are attributed to the Pd^2+^ [16]. Obviously, Pd^0^ and Pd^2+^ coexisted on the catalyst surface, based on the analysis results. The percentages of Pd^2+^ species on the Au-Pd/TiO_2_ catalysts were obtained by XPS fitting areas Pd^2+^/(Pd^2+^+Pd^0^). The contents of Pd^2+^ on the catalyst surface were arranged in the following order: ATR (55.4%) > ATB (48.2%) > ATA (34.8%). It is generally accepted that the formation of Pd^2+^ species on the catalyst surface is closely related to the drying and calcination processes [17]. Nevertheless, the higher ratios of Pd^2+^ on the ATR and ATB indicated that the TiO_2_ carrier also played an essential role in promoting the formation of Pd^2+^, which could supply oxygen to assist the production of Pd^2+^. The existence of Pd^2+^ species further demonstrates that some Pd cannot alloy with Au; this phenomenon has been reported in the similar Au-Pd/CeO_2_ catalyst [18].

The XPS spectra of O1s on the ATA, ATB, and ATR catalysts are exhibited in Fig. 2.3. As reported, the O1s peak can be fitted into two sub-peaks. The sub-band at the lower banding energy (529.1 eV) can be attributed to the lattice oxygen (O_β_) and the sub-band at the higher binding energy (531.0 eV) can be assigned to the surface adsorbed oxygen (O_α_). In the traditional catalytic oxidation reactions, the surface adsorbed oxygen usually displays higher reactivity than lattice oxygen, due to its higher mobility [19]. Hence, the O_α_ ratios on these three Au-Pd/TiO_2_ catalysts were calculated by XPS fitting areas O_α_/(O_α_+O_β_). It was found that the O_α_ ratio on the ATR (43.8%) is higher than the O_α_ ratio on the ATB (38.7%) and ATA (20.2%). The O_α_ ratio can also be applied to estimate the contents of oxygen vacancies on the catalyst surface, which play a key role in stabilizing the Au-Pd nanoparticles on the catalyst and promoting the catalytic activity. The O_α_ ratios are consistent with the Pd^2+^ concentrations on the catalyst surface. Figure 2.4 presents the Ti2p XPS spectra. The peaks are centered at about 464.2 and 458.4 eV; this could be attributed to the Ti 2p_1/2_ and Ti 2p_3/2_ of Ti^4+^ in TiO_2_, respectively, indicating that Ti was in the oxidation state of +4 [20].

The surface atomic concentrations obtained from the XPS characterization results are summarized in Table 2. Compared with the Au-Pd bulk compositions determined by ICP-AES, it can be found that the content of Au on the surface of the Au-Pd/TiO_2_ catalysts is lower than that in the corresponding bulk. The concentrations of Pd on the catalyst surface also exhibited a similar trend, except for the Pd on the ATR. Based on the determined Au and Pd concentrations on the catalyst’s surface, the molar ratios of Au/Pd were calculated and ranked by ATA > ATB > ATR. These values were found lower than the nominal and bulk Au/Pd values, which suggests that the interaction between metal nanoparticles and TiO_2_ is closely related to the carrier forms.

**Table 2:** Surface element composition on the Au-Pd/TiO_2_ catalysts

Catalysts	Surface content (atomic %)				Au/Pd molar ratio
	Au 4f	Pd3d	O1s	Ti3d	
ATA	0.32	0.36	69.76	29.56	0.48
ATB	0.24	0.41	66.11	33.24	0.42
ATR	0.36	0.65	81.46	17.54	0.30

The surface element concentration was determined by XPS

**Fig. 3:**
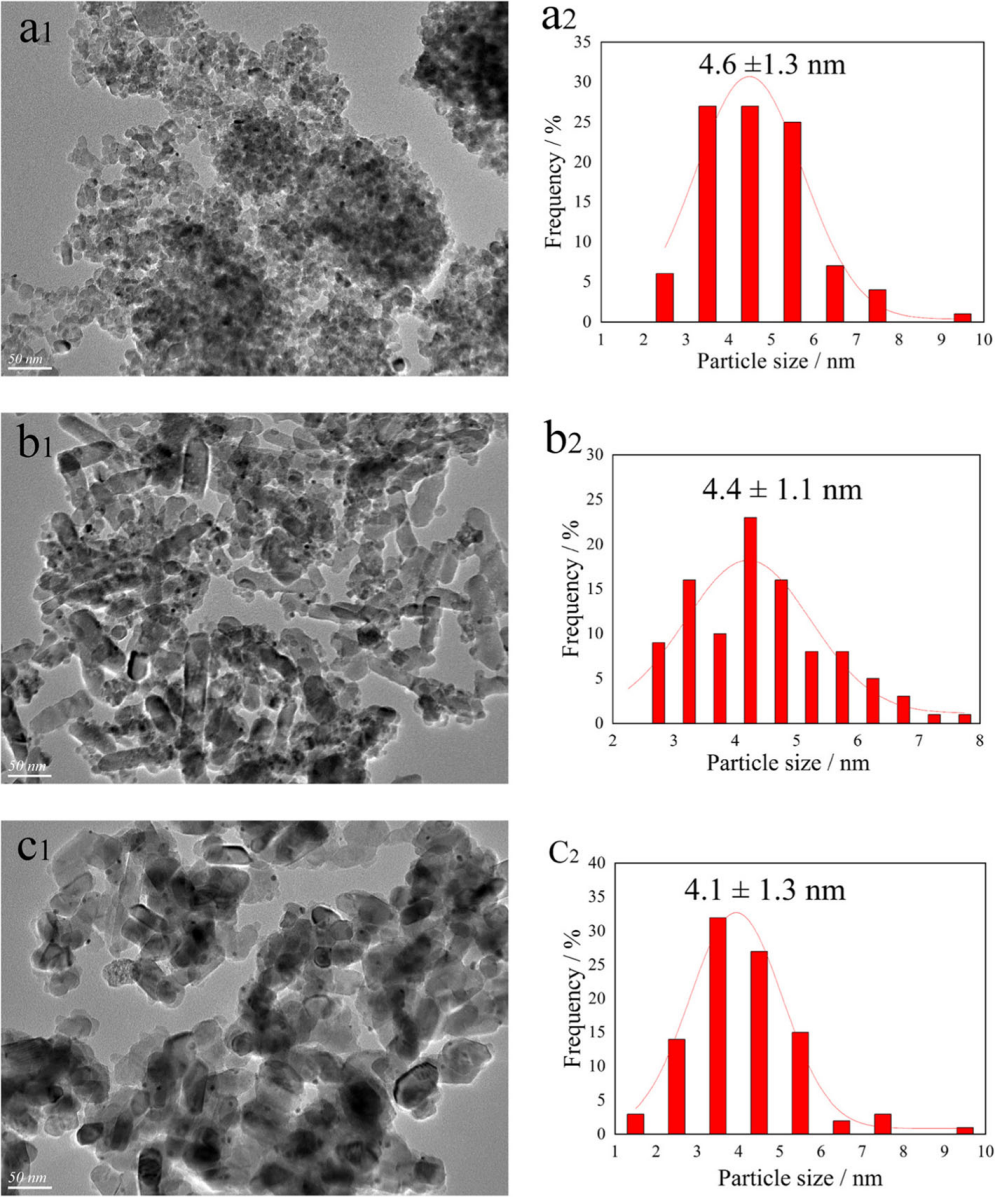
TEM images and Au-Pd particle size distribution histograms of the ATA (**a**_**1**_, **a**_**2**_), ATB (**b**_**1**_, **b**_**2**_), and ATR (**c**_**1**_, **c**_**2**_) catalysts

TEM was conducted to investigate the morphology of the catalysts and the size distributions of Au-Pd nanoparticles on the catalyst surface. The corresponding TEM images and histograms of Au-Pd distributions are presented in Fig. 3. It is noteworthy that more than 100 nanoparticles were measured to calculate the average particle size. As exhibited in Fig. 3a, the carrier agglomeration phenomenon observed on the ATA catalyst and the Au-Pd nanoparticles size distribution could be described using a lognormal distribution, with a mean size of approximately 4.6 nm. Meanwhile, it was found that brookite TiO_2_ presented a rod-like shape and the average Au-Pd nanoparticle size on the ATB was smaller than the nanoparticle size on ATA. This result was consistent with Dai’s report [11]. For ATR catalyst, it got the most uniform dispersion of Au-Pd nanoparticles on the catalyst surface, and the smallest average particle size (4.1 nm) was obtained. The TEM results indicated that the mean particle size and the particle size distribution are strongly related to the properties and the forms of the catalyst carrier.

### Catalytic Activity Measurement

**Fig. 4:**
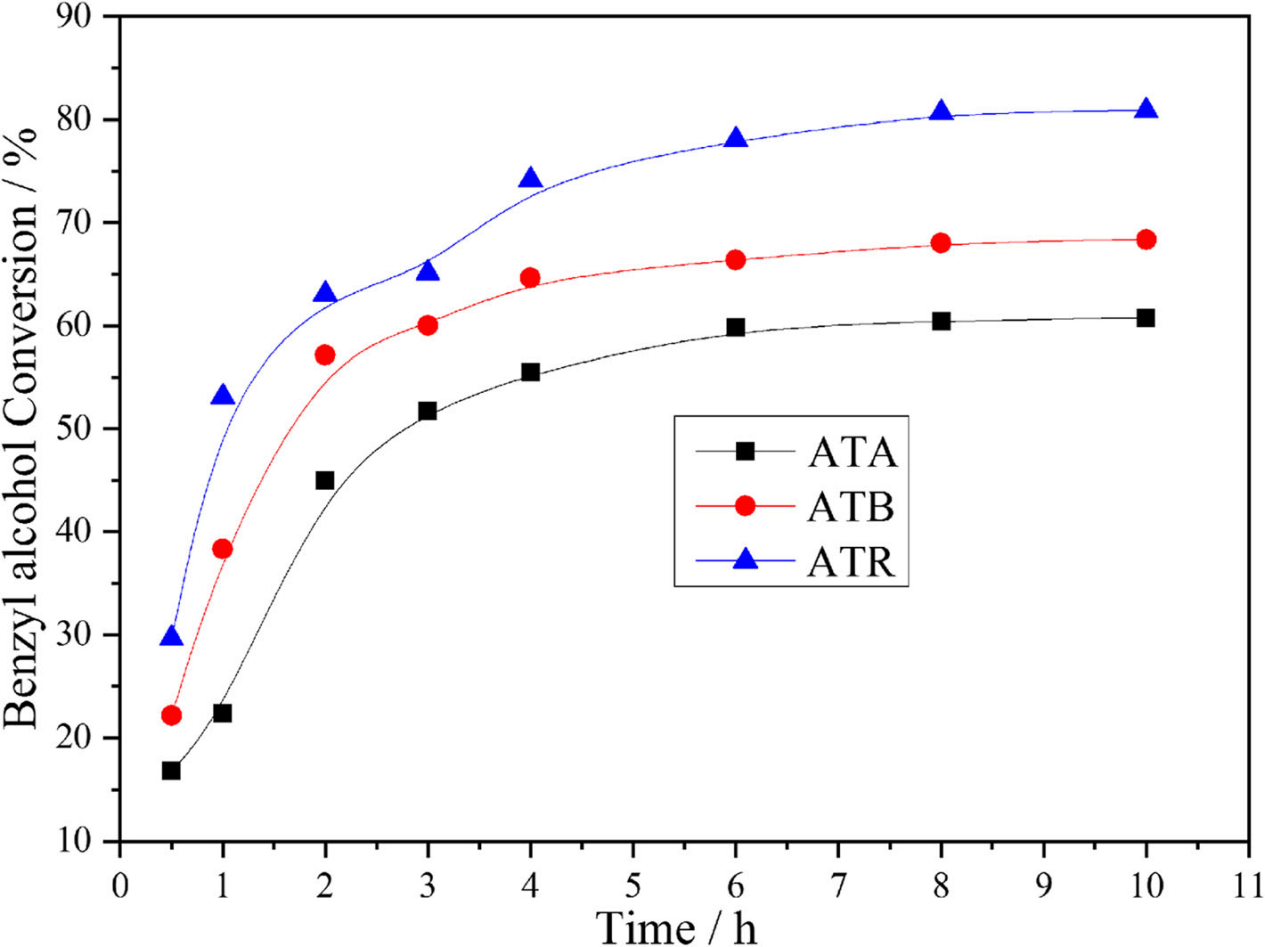
Benzyl alcohol conversion as a function of time achieved on the ATA, ATB, and ATR

**Fig. 5:**
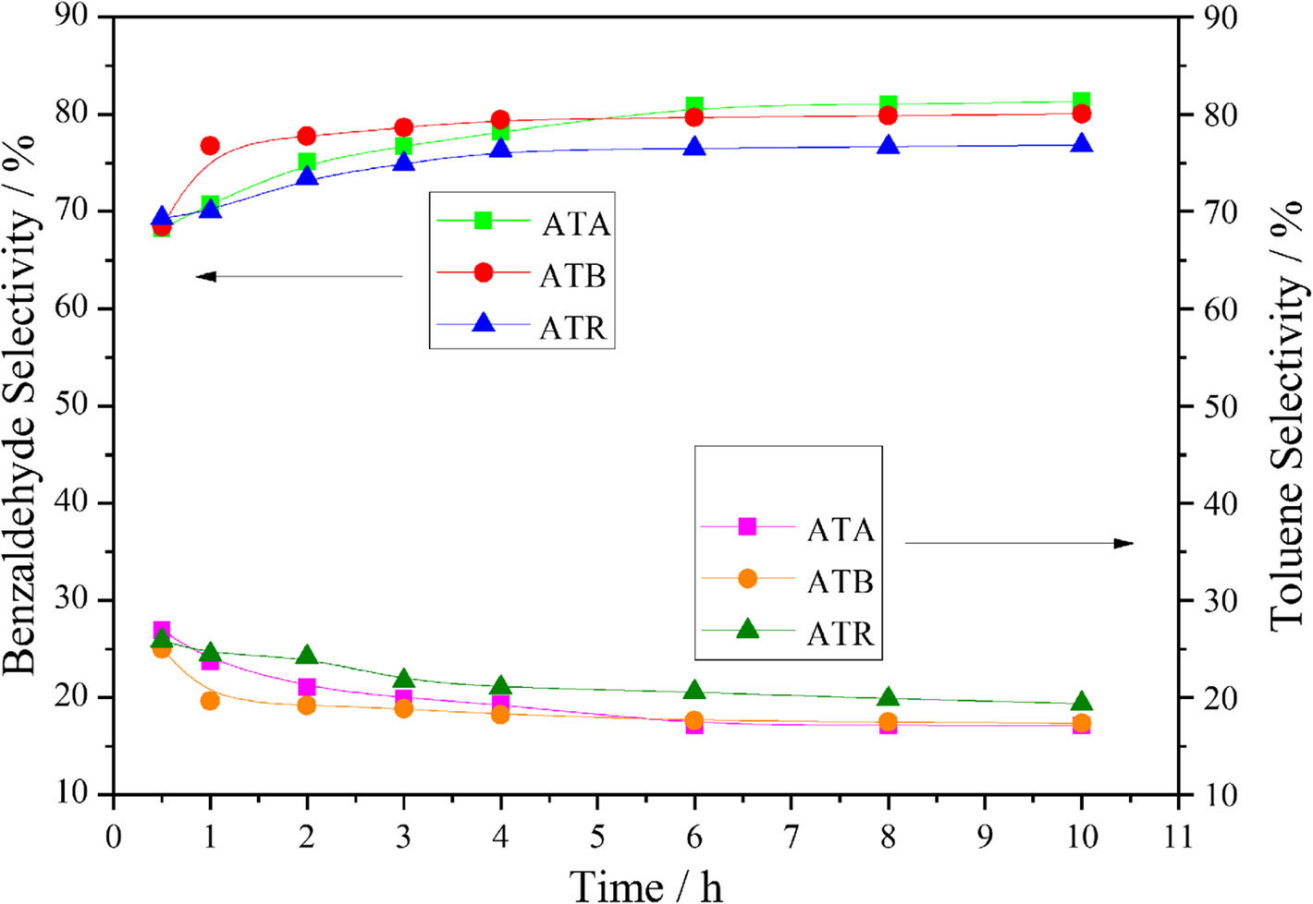
Benzaldehyde and toluene selectivities as a function of time obtained on the ATA, ATB, and ATR

Au-Pd nanoparticles supported on different forms of TiO_2_ were investigated for the benzyl alcohol oxidation, with 0.3 MPa pure oxygen at 120 °C under the solvent-free condition. The corresponding results are shown in Fig. 4. As can be seen from the figure, the benzyl alcohol conversion on the ATR catalyst achieved about 65.11% after 3 h of reaction. However, only 60.01% and 51.75% benzyl alcohol conversions could be obtained over the ATB and ATA catalysts under the same condition. Besides, it was found that the benzyl alcohol conversion could be arranged in the following order throughout the investigation period: ATR > ATB > ATA. According to the XPS characterization results, we can find that the O_α_ and Pd^2+^ ratios also exhibited similar trends, which indicated that the O_α_ and Pd^2+^ ratios play a key role in determining the catalytic performance. Furthermore, the TEM result suggested that the smaller Au-Pd particle size was obtained on the ATR, which is also helpful for promoting the catalytic activity. As we all know, the main products for the benzyl alcohol oxidation reaction are benzaldehyde and toluene, and the remaining by-products include benzene, benzoic acid, and benzyl benzoate. All these products were detected during our experiments and the typical case is listed in Table 3, which is consistent with the published literatures of benzyl alcohol oxidation over Au-Pd immobilized catalysts [21, 22]. Meanwhile, it could be found that ATR catalyst exhibited higher selectivity to toluene and lower selectivity to benzaldehyde compared with ATA and ATB catalysts during the whole reaction process (Fig. 5).

**Table 3:** Catalytic oxidation of benzyl alcohol over Au-Pd/TiO_2_ catalysts in the absence of solvent

Samples	Conversion	Selectivity/%					
	%	Benzene	Toluene	Benzoic acid	Benzaldehyde	Benzyl benzoate	others
ATA	51.75	0.62	19.94	0.43	76.71	2.11	0.19
ATB	60.01	0.47	18.24	0.64	78.53	1.86	0.26
ATR	65.11	0.37	21.75	0.55	74.91	2.09	0.24

Reaction condition: benzyl alcohol 15 mL, catalyst 50 mg, O_2_ 0.3 MPa, 120 °C 3 h

**Fig. 6:**
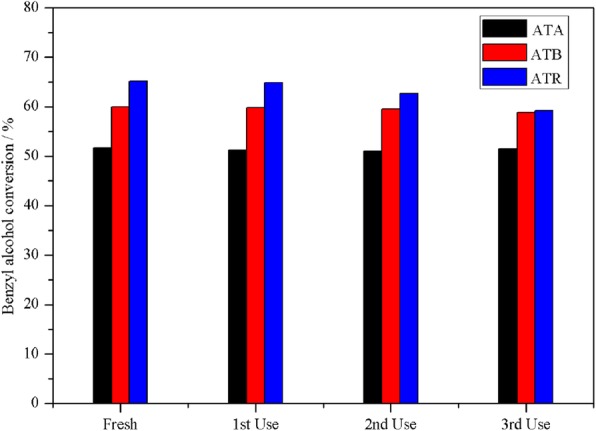
The reuse of the ATA, ATB, and ATR catalysts in benzyl alcohol oxidation under solvent-free

To further investigate the stability of the Au-Pd/TiO_2_ catalysts in benzyl alcohol oxidation, the catalytic performances of the ATA, ATB, and ATR catalysts were studied with repeated usage. The corresponding results are provided in Fig. 6. After each activity evaluation, the catalyst was separated from the mixture solution by centrifugation, then washed by acetone, and heated at 80 °C for 16 h. It was found that the ATA and ATB samples showed higher catalytic stability, compared with the ATR sample. The benzyl alcohol conversion in the 1st, 2nd, and 3rd recycle was 51.28%, 51.06%, and 51.49%, respectively, for the ATA catalyst, and 59.78%, 59.54%, 58.76%, respectively, for the ATB sample. However, the benzyl alcohol conversion over the ATR sample exhibited a significant decline after each cycle; the catalytic activity decreased from the initial 65.11% to the final 59.22%, which might be due to Pd poisoning. The Pd was poisoned when the catalyst was saturated with the products during the reaction. It is widely reported that Pd-based catalyst becomes easily deactivated due to the desorption problem of aldehyde products on the catalyst surface [22–25]. In our case, ATR was found to contain the highest concentration of Pd (0.65 atomic%) on the catalyst surface from XPS measurements.

## Conclusion

In conclusion, Au-Pd nanoparticles with a molar ratio of 1:1 were deposited on the different forms of TiO_2_ through the deposition-precipitation method. Benzyl alcohol oxidation was selected as the probe reaction to investigate the catalytic activities in the absence of solvents. Based on the characterization and activity evaluation results, it could be found that the catalytic activity of the Au-Pd/TiO_2_ catalyst was firmly related to the TiO_2_ form. Characterization results of the XPS and TEM suggested that ATR surface contained larger O_α_ and Pd^2+^ concentrations together with the smaller Au-Pd nanoparticle size compared with ATB and ATA catalysts, which played critical roles in obtaining the high benzyl alcohol conversion. However, ATR catalyst exhibited lower catalytic stability compared with the ATA and ATB catalysts, which might be related to the coverage of larger amount of aldehyde products on the surface during the reaction process.

## Data Availability

All the data are fully available without restrictions.

## Abbreviations

ATA: Au-Pd/TiO_2_-anatase
ATB: Au-Pd/TiO_2_-brookite
ATR: Au-Pd/TiO_2_-rutile
FID: Flame ionization detector
GC: Gas chromatography
ICP-AES: Inductively coupled plasma atomic emission spectrometry
TBD: Titanium bis (ammonium lactate) dihydroxide
TEM: Transmission electron microscope
TiO_2_-A: TiO_2_-anatase
TiO_2_-B: TiO_2_-brookite
TiO_2_-R: TiO_2_-rutile
XPS: X-ray photoelectron spectroscopy
XRD: X-ray powder diffraction

## References

[1] Hutchings GJ (1985). Vapor phase hydrochlorination of acetylene: correlation of catalytic activity of supported metal chloride catalysts. J Catal.

[2] Haruta M, Kobayashi T, Sano H, Yamada N (1987). Novel gold catalysts for the oxidation of carbon monoxide at a temperature far below 0^o^C. Chem Lett.

[3] Burch R (2006). Gold catalysts for pure hydrogen production in the water-gas shift reaction: activity, structure and reaction mechanism. Phys Chem Chem Phys.

[4] Edwards JK, Hutchings GJ (2008). Palladium and gold-palladium catalysts for the direct synthesis of hydrogen peroxide. Angew Chem Int Ed.

[5] Bus E, Prins R, Bokhoven JA (2007). Origin of the cluster-size effect in the hydrogenation of cinnamaldehyde over supported Au catalysts. Catal Commun.

[6] Enache D, Edwards J, Landon P, Espriu B, Carley AF, Herzing AA, Watanabe M, Kiely CJ, Knight DW, Hutchings GJ (2006). Solvent-free oxidation of primary alcohols to aldehydes using Au-Pd/TiO_2_ catalysts. Science.

[7] Khawaji M, Chadwick D (2017). Au-Pd bimetallic nanoparticles immobilised on titanate nanotubes: a highly active catalyst for selective oxidation. ChemCatChem.

[8] Sun J, Han Y, Fu H, Qu X, Xu Z, Zheng S (2017). Au@ Pd/TiO_2_ with atomically dispersed Pd as highly active catalyst for solvent-free aerobic oxidation of benzyl alcohol. Chem Eng J.

[9] Hong Y, Jing X, Huang J, Sun D, Wubah T, Yang F, Du M, Li Q (2014). Biosynthesized bimetallic Au-Pd nanoparticles supported on TiO_2_ for solvent-free oxidation of benzyl alcohol. ACS Sustain Chem Eng.

[10] Liu L, Zhao H, Andino JM, Li Y (2012). Photocatalytic CO_2_ reduction with H_2_O on TiO_2_ nanocrystals: comparison of anatase, rutile, and brookite polymorphs and exploration of surface chemistry. ACS Catal.

[11] Yan W, Chen B, Mahurin SM, Schwartz V, Mullins DR, Lupini AR, Pennycook SJ, Dai S, Overbury SH (2005). Preparation and comparison of supported gold nanocatalysts on anatase, brookite, rutile, and P25 polymorphs of TiO_2_ for catalytic oxidation of CO. J Phys Chem B.

[12] Yao X, Zhao R, Chen L, Du J, Tao C, Yang F, Dong L (2017). Selective catalytic reduction of NO_x_ by NH_3_ over CeO_2_ supported on TiO_2_: comparison of anatase, brookite, and rutile. Appl Catal B Environ.

[13] Kandiel TA, Feldhoff A, Robben L, Dillert R, Bahnemann DW (2010). Tailored titanium dioxide nanomaterials: anatase nanoparticles and brookite nanorods as highly active photocatalysts. Chem Mater.

[14] Jiang P, Porsgaard S, Borondics F, Köber M, Caballero A, Bluhm H, Besenbacher F, Salmeron M (2010). Room-temperature reaction of oxygen with gold: an in situ ambient-pressure X-ray photoelectron spectroscopy investigation. J Am Chem Soc.

[15] Yarulin AE, Crespo-Quesada RM, Egorova EV, Kiwi-Minsker LL (2012). Structure sensitivity of selective acetylene hydrogenation over the catalysts with shape-controlled palladium nanoparticles. Kinet Catal.

[16] Desforges A, Backov R, Deleuze H, Mondain-Monval O (2005). Generation of palladium nanoparticles within macrocellular polymeric supports: application to heterogeneous catalysis of the Suzuki-Miyaura coupling reaction. Adv Funct Mater.

[17] Pecchi G, Reyes P, Concha I, Fierro J (1998). Methane combustion on Pd/SiO_2_ sol gel catalysts. J Catal.

[18] Khawaji M, Chadwick D (2018). Au-Pd NPs immobilised on nanostructured ceria and titania: impact of support morphology on the catalytic activity for selective oxidation. Catal Sci Technol.

[19] Shan W, Liu F, He H, Shi X, Zhang C (2012). An environmentally-benign CeO_2_-TiO_2_ catalyst for the selective catalytic reduction of NO_x_ with NH_3_ in simulated diesel exhaust. Catal Today.

[20] Dimitratos N, Lopez-Sanchez JA, Morgan D, Carley AF, Tiruvalam R, Kiely CJ, Bethell D, Hutchings GJ (2009). Solvent-free oxidation of benzyl alcohol using Au-Pd catalysts prepared by sol immobilisation. Phys Chem Chem Phys.

[21] Chen Y, Lim H, Tang Q, Gao Y, Sun T, Yan Q, Yang Y (2010). Solvent-free aerobic oxidation of benzyl alcohol over Pd monometallic and Au-Pd bimetallic catalysts supported on SBA-16 mesoporous molecular sieves. Appl Catal A-Gen.

[22] Naughton J, Lee AF, Thompson S, Vinod CP, Wilson K (2010). Reactivity of crotonaldehyde and propene over Au/Pd (111) surfaces. Phys Chem Chem Phys.

[23] Bowker M, Cookson L, Bhantoo J, Carley A, Hayden E, Gilbert L, Morgan C, Counsell J, Yaseneva P (2011). The decarbonylation of acetaldehyde on Pd crystals and on supported catalysts. Appl Catal A Gen.

[24] Lee AF, Naughton JN, Liu Z, Wilson K (2012). High-pressure XPS of crotyl alcohol selective oxidation over metallic and oxidized Pd (111). ACS Catal.

[25] Parlett CMA, Durndell LJ, Machado A, Cibin G, Bruce DW, Hondow NS, Wilson K, Lee AF (2014). Alumina-grafted SBA-15 as a high performance support for Pd-catalysed cinnamyl alcohol selective oxidation. Catal Today.

